# Citrate Stabilizes Hydroxylapatite Precursors: Implications for Bone Mineralization

**DOI:** 10.1021/acsbiomaterials.1c00196

**Published:** 2021-05-11

**Authors:** Encarnacion Ruiz-Agudo, Cristina Ruiz-Agudo, Fulvio Di Lorenzo, Pedro Alvarez-Lloret, Aurelia Ibañez-Velasco, Carlos Rodriguez-Navarro

**Affiliations:** † Department of Mineralogy and Petrology, University of Granada, Fuentenueva s/n, Granada 18071, Spain; ‡ Physical Chemistry, Department of Chemistry, University of Konstanz, Universitätsstraße 10, Konstanz 78457, Germany; § Institute of Geological Sciences, University of Bern, Baltzerstrasse 3, Bern CH-3012, Switzerland; ∥ Department of Geology, University of Oviedo, C/Jesús Arias de Velasco s/n, Oviedo 33005, Spain

**Keywords:** citrate, calcium phosphate, liquid-like precursor, prenucleation species, amorphous calcium phosphate

## Abstract

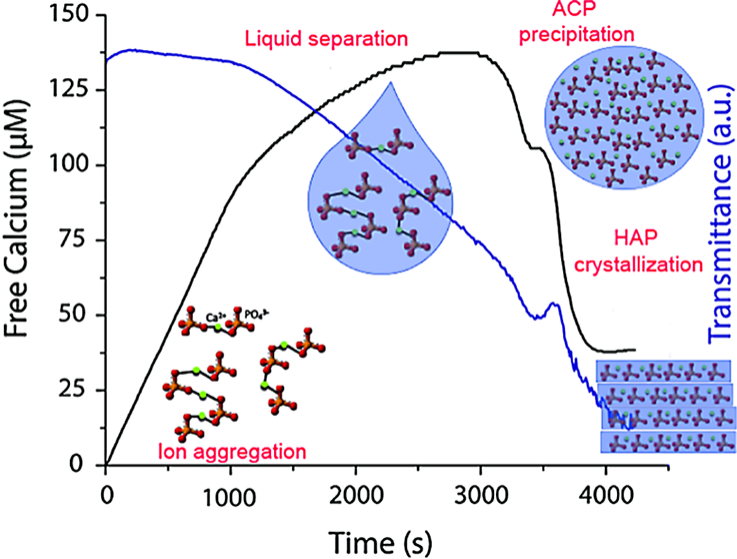

Mineralization of hydroxylapatite (HAp), the main inorganic phase in bone, follows nonclassical crystallization routes involving metastable precursors and is strongly influenced by organic macromolecules. However, the effect of small organic molecules such as citrate on the formation of HAp is not well constrained. Using potentiometric titration experiments and titration calorimetry, in combination with a multianalytical approach, we show that citrate stabilizes prenucleation species as well as a liquid-like calcium phosphate precursor formed before any solid phase nucleates in the system. The stabilization of a liquid-like precursor phase could facilitate infiltration into the cavities of the collagen fibrils during bone mineralization, explaining the enhancement of collagen-mediated mineralization by citrate reported in previous studies. Hence, citrate can influence bone mineralization way before any solid phase (amorphous or crystalline) is formed. We also show that HAp formation after amorphous calcium phosphate (ACP) in the absence and presence of citrate results in nanoplates of about 5–12 nm thick, elongated along the *c* axis. Such nanoplates are made up of HAp nanocrystallites with a preferred *c* axis orientation and with interspersed ACP. The nanoplatelet morphology, size, and preferred crystallographic orientation, remarkably similar to those of bone HAp nanocrystals, appear to be an intrinsic feature of HAp formed from an amorphous precursor. Our results challenge current models for HAp mineralization in bone and the role of citrate, offering new clues to help answer the long-standing question as to why natural evolution favored HAp as the mineral phase in bone.

## 1 Introduction

Many living organisms take advantage of nonclassical crystallization routes (e.g., formation of amorphous precursors or growth by the nanoparticle assembly) to build complex hierarchical mineral microstructures.^1−3^ Defective hydroxylapatite (Ca_10_(PO_4_)_6_(OH)_2_; HAp), or bioapatite, the biomineral composing mammalian bone and teeth, is one of the most prominent examples. It is long known that HAp biomineralization entails the formation of intermediate states such as amorphous precursors,^4^ although the exact conversion mechanism to crystalline HAp is still under debate. Also, it has been proposed that prenucleation clusters are involved in HAp formation.^5−7^ These prenucleation species could be considered as Posner’s clusters, first postulated in 1975,^8^ and later found in simulated body fluids using an intensity-enhanced dynamic light scattering (DLS) technique.^9^ These clusters were envisaged as the solution precursors to amorphous calcium phosphate (ACP).^9^ In addition, the assumed fluidic character of calcium phosphate precursors is critical for achieving optimal collagen mineralization.^2,10^ However, despite the significant experimental evidence of “nonclassical” processes during bone biomineralization, the exact steps behind the biological HAp formation are largely unknown.

An additional issue arises from the fact that calcium phosphate biominerals (e.g., bone and dentine) can be considered heterogeneous biocomposites, formed as well by an organic phase consisting of collagen, noncollagenous proteins (NCPs), water, and other components.^11^ Organic macromolecules are commonly suggested to be the main actors responsible for the liquid-like character of the ACP precursor,^2^ and most studies have focused on their role on calcium phosphate formation.^7,12,13^ Among the “other components” in the organic fraction, citrate is one of the most relevant molecules, comprising ∼2% weight of bone, which represents 80% of total citrate in the body.^14^ All “osteovertebrates” show this high citrate level, which suggests that it is also a key component of bone and reflects its necessary participation in the mineralization process.^14^ Furthermore, citrate is commonly used as a drug for osteoporosis treatment due to its ability of enhancing collagen mineralization.^15^ In spite of this, its role during bone formation, regeneration, and bone disorders remains largely discussed and unaddressed. Early research efforts aimed at unraveling the role of citrate in bone formation and other related issues diminished since the beginning of 1975.^14^ However, in the last decade, renewed interest on the role of citrate in collagen mineralization has inspired much research.^12,15−22^

Recent studies have shown the existence of two major pools of citrate in bone: HAp-associated citrate and collagen-bound citrate.^14^ The incorporation of citrate between mineral nanoplatelets has been claimed to control HAp crystallinity, crystal size, morphology, and preferred orientation,^23^ an effect that is key to achieve the superior mechanical properties of bone and resorption during bone remodeling.^19,24^ Also, it has been hypothesized that the role that citrate ions play in bone resorption and/or ossification is simply related to their ability to form complexes with calcium ions in the surrounding body fluid.^25^ More recently, citrate has also been shown to influence different stages of HAp formation, by first directing its growth via an amorphous solid precursor (ACP)^12,13^ and subsequently by limiting the growth of HAp nanocrystals by an oriented aggregation mechanism.^20^ Delgado-López et al.^22^ proposed that citrate facilitates the infiltration of calcium phosphate into collagen fibrils during bone mineralization, an effect that has been recently ascribed to the fact that collagen-bound citrate reduces the interfacial energy between ACP and collagen, thus facilitating the heterogeneous nucleation of ACP onto collagen.^15^ Overall, these recent studies agree that citrate promotes collagen mineralization, although the exact mechanism for this effect remains highly speculative.

The aim of this research is to elucidate the effect of citrate as a controlling agent during the early stages of calcium phosphate crystallization and to highlight its possible role in biological HAp formation. Here, we provide direct experimental evidence of the existence of a liquid-like precursor to HAp formation that, together with other early-stage species (i.e., prenucleation clusters), is stabilized in the presence of citrate. These results increase our understanding on the physical–chemical processes involved in the modulation of calcium phosphate nucleation and early growth stages by citrate, offering new insights into bone mineralization mechanisms, where citrate plays a key role much earlier than previously thought. We also show that citrate is not critical to modulate the platelike morphology and extreme thinness of HAp nanocrystals as it is currently accepted. Such features are also observed in HAp formed after an amorphous precursor phase in the absence of citrate.

## 2 Results and Discussion

### 2.1 Evolution of the Pure System in the Prenucleation Regime

**Figure 1:**
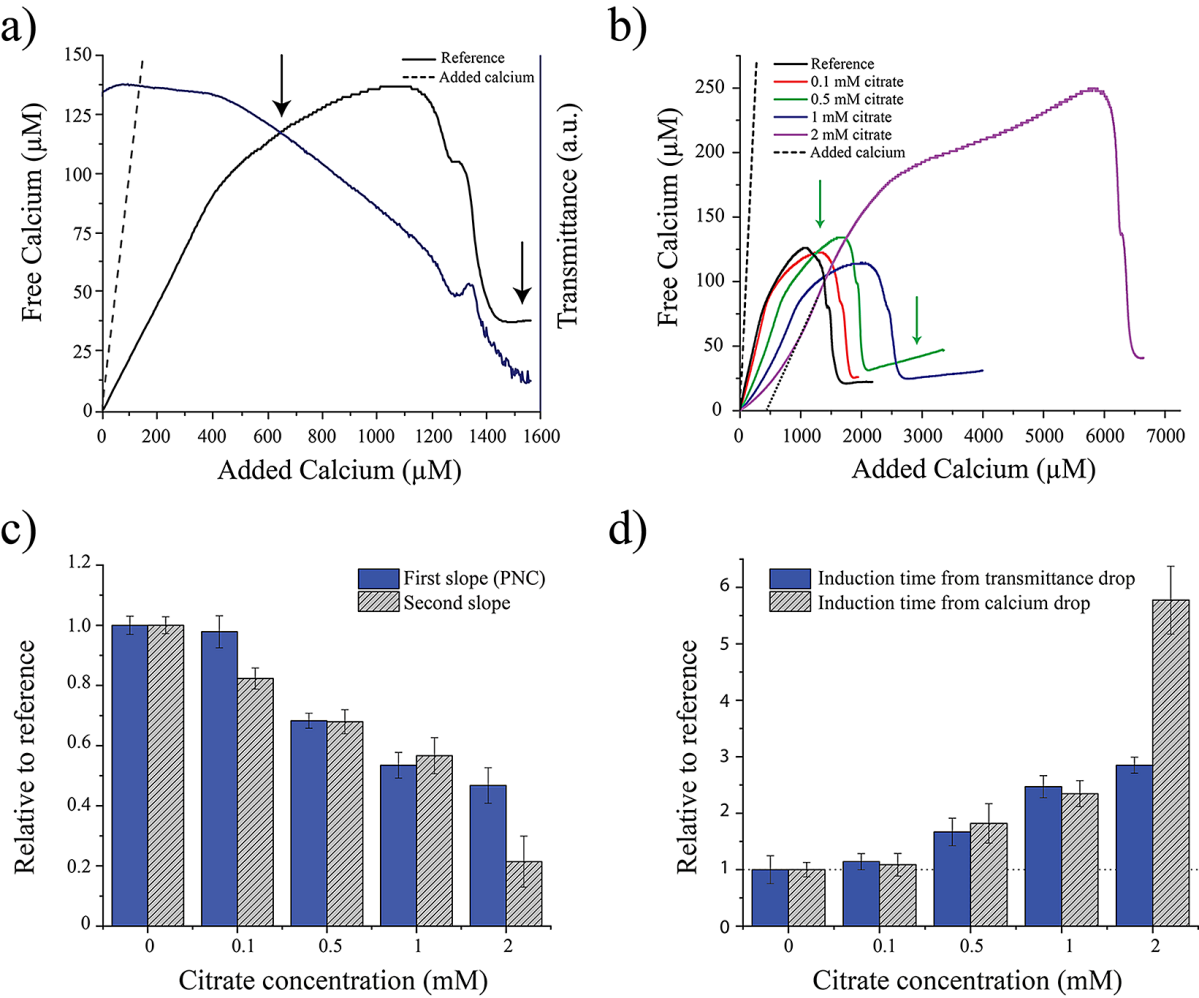
(a) Typical time development of free Ca^2+^ concentration in control (i.e., no citrate in the phosphate solution) runs at pH 8.0 (black line, calcium addition rate = 2.4 μmol/min). The dashed line depicts the Ca concentration added to the vessel. Evolution of transmittance is also represented (blue line). (b) Time-dependent progression of the amount of free Ca^2+^ concentration detected during the addition of CaCl_2_ into phosphate buffer (pH 8) containing different amounts of citrate (color curves), as compared to the reference without additives (black curve). The dotted line represents the extrapolation of the linear regime of the free calcium curve at a concentration of 2 mM citrate and depicts a binding of 0.06 Ca^2+^ per COOH^–^. The arrows mark where aliquots were drawn for transmission electron microscopy (TEM) analysis. The bar plots illustrate the effect of added citrate on (c) the slope of *t*-dependent free Ca^2+^ for the two different regimes found before the calcium drop and (d) the transmittance and calcium drop. The results are given as relative changes to the reference experiments, that is, as quotients of the respective values determined in the presence and absence of citrate. Error bars show standard deviation (SD).

The in situ monitoring of free calcium concentration and transmittance (Figure 1a, control run) during potentiometric calcium titration experiments (see Section 4) systematically shows that the free calcium concentration measured in solution increases linearly but is lower than the calcium added (dashed line). Note that while ion-selective electrode measurements based on calibrations in water give calcium activities, calibrations in NaCl solutions such as those performed here lead to actual concentrations (for a detailed analysis of ion activity treatment in titration experiments, see ref (26)). This behavior has been observed for numerous sparingly soluble salts (e.g., calcium carbonate, calcium oxalate, and barium sulfate) and has been related to the formation of stable prenucleation associates in solution.^5^ Moreover, this agrees with previous observations,^6,7^ suggesting the existence of stable clusters prior to the formation of a solid Ca-phosphate phase. As mentioned above, these prenucleation species could be considered to be Posner’s clusters, which are considered as the solution precursor to ACP formation.^8,9^

Subsequently, the calcium plots deviate from the initial linear trend, showing a sudden change in the slope (Figure 1). Similar findings have been reported during calcium phosphate formation in the presence of osteopontin, an NCP found in mammalian bone and teeth, and are ascribed to the formation of a (liquid-like) intermediate precursor to the final solid phase.^7^ In our case, the decrease in the slope of the free calcium coincides with a change in the slope of the transmittance curve (Figure 1a), which in the early stages of the experiment remains approximately constant (with a value close to zero), but decreases from this point on. A direct comparison of our titration data with recent works^7^ suggests the formation of a Ca–P-bearing liquid-like intermediate, in our case even in the absence of additives. However, further analyses were performed to confirm this hypothesis (see below).

Experiments performed at different calcium addition rates (Figure S1) suggest that the observation of this intermediate Ca–P-bearing phase in the absence of any additive relates to a kinetic effect associated with the much faster rise in supersaturation in our experiments than in previous experiments.^7^ A liquid–liquid phase separation may occur in a crystallizing system when supersaturation is increased fast enough so that the system reaches the binodal or even the spinodal regime, without permitting nucleation when going past the region where nucleation of a (crystalline or amorphous) solid phase can occur.^27,28^ Liquid–liquid phase separation has been suggested to occur upon the aggregation of prenucleation ion associates such as those mentioned above. From a molecular point of view, it has been hypothesized that upon reaching a critical size, the dynamics of the structural rearrangement of the prenucleation clusters (PNC) slows down, and they become dense-liquid nanodroplets. Aggregation and coalescence of the latter can result in liquid droplets of several hundreds of nanometers.^29^

#### 2.1.1 Further Evidence for a Liquid–Liquid Separation

**Figure 2:**
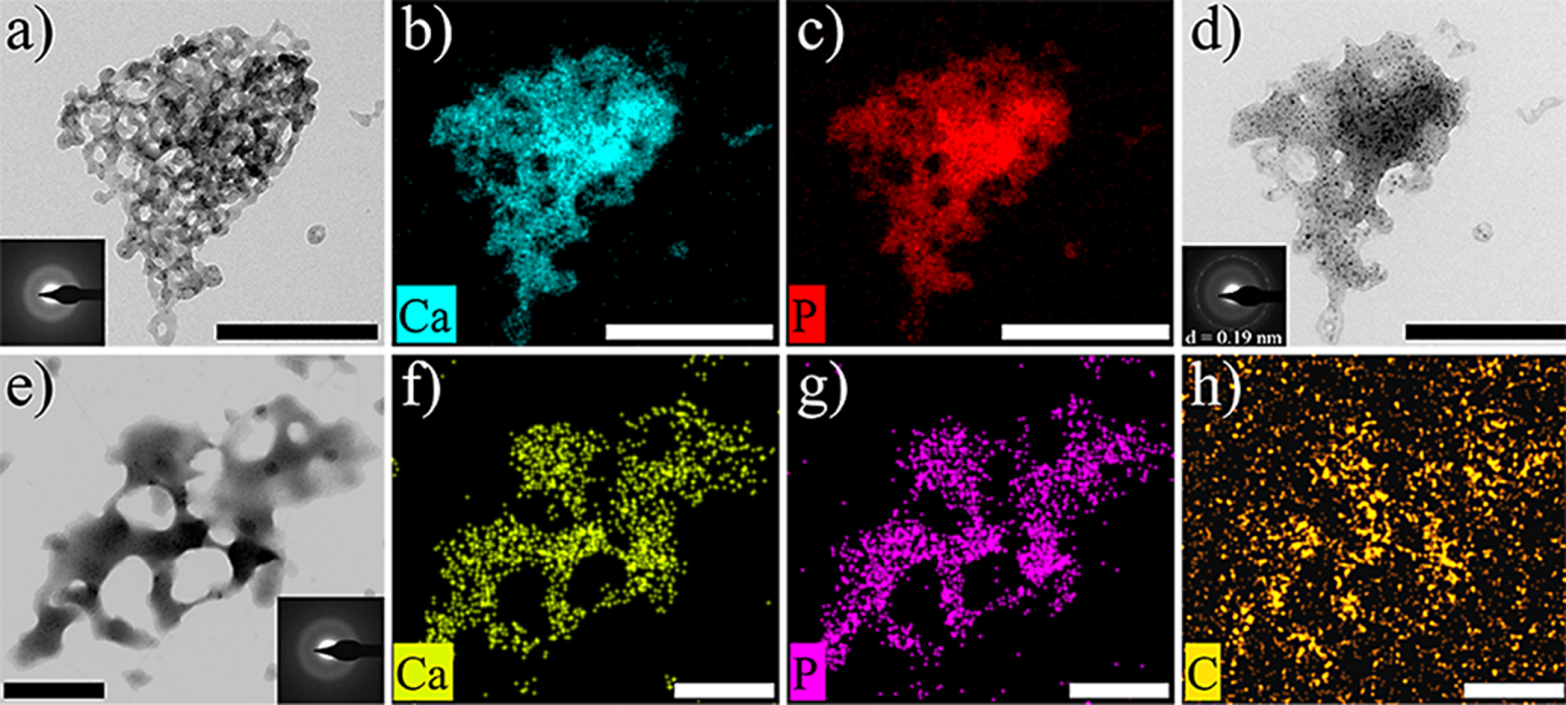
(a) TEM images of precipitates formed in control runs (i.e., no citrate in the phosphate solution) in titration experiments performed at pH 8 (ca. 1600 s, see the first black arrow in Figure 1b). The SAED pattern in the inset demonstrates its amorphous character. (b, c) Calcium and phosphorus elemental EDS map of the precipitate in (a). (d) TEM image of the same precipitates in (a) upon irradiation under the electron beam for ca. 10 min. The SAED pattern (inset) shows diffraction rings that are in agreement with beam-induced HAp crystallization. (e) TEM image of calcium phosphate precipitates resembling an emulsion (in the dry state), obtained upon the sampling of the reaction media at 1600 s (i.e., prenucleation stage, first green arrow in Figure 1b). This structure is formed in the presence of 0.5 mM citrate at pH 8. The inset shows the SAED pattern of this amorphous structure. (b–d) Calcium, phosphorus, and carbon elemental EDS map of the precipitate in (e). Scale bar = 200 nm.

Isothermal pseudotitration calorimetry (ITC) data support the occurrence of a first-order phase transition prior to the formation of the first solid phase in the system, consistent with the occurrence of a liquid-like precursor phase as described above. A first-order transition is defined as one in which a discontinuity occurs in the first derivative of the free energy, which corresponds to entropy, volume, or enthalpy. Therefore, this transition can be identified by the apparent discontinuity seen in the enthalpy versus added calcium curve under the same conditions, as identified in the reference titration experiments (between 1000 and 1200 s) (Figure S2). Interestingly, as it has been observed for other systems,^30^ this discontinuity is endothermic (i.e., the slope of the enthalpy plot becomes less negative) and thus the liquid–liquid separation process must be driven by an entropy increase. Desolvation associated with the separation of a liquid phase would represent a major contribution to such positive entropy change. Further evidence for the formation of a dense liquid phase is provided from TEM observations of aliquots quenched ca. 1600 s (first arrow in Figure 1a) in control runs (Figure 2a–d). Amorphous structures with irregular morphologies and sizes (up to several hundreds of nm) are seen in samples drawn from the reaction solution at this stage and quenched in ethanol. These structures resemble the aggregates of nanoparticles (ca. 20 nm in size) that tend to develop spherical morphologies, connected with necklike bridges. The solids shown in Figure 2 are very similar to those observed by Rieger et al.^31^ during CaCO_3_ formation using cryo-TEM and interpreted to be a solid remnant of a liquid-like structure (precursor of amorphous calcium carbonate). These structures are highly electron beam sensitive (Figure 2a vs Figure 2d), and once irradiated, direct HAp crystallization can be induced within them, as indicated by the appearance of Debye rings in the selected area electron diffraction (SAED) pattern with a *d*-spacing of 0.19 nm, corresponding to the 222 Bragg reflections of HAp (see the inset in Figure 2d).

**Figure 3:**
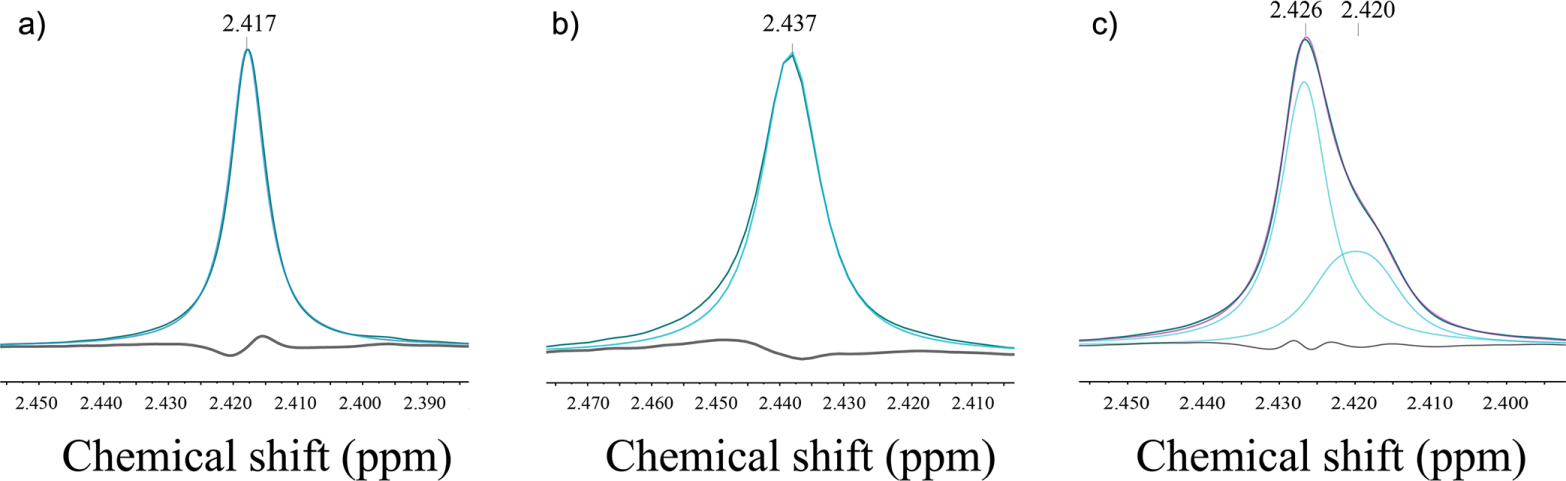
One-dimensional ^31^P NMR spectrum of (a) 20 mM phosphate buffer (no citrate added), pH 8.0; (b) sample drawn from solution during titration experiments before the first phase transition (i.e., first slope of the free-Ca curve); (c) sample drawn from titration experiments after the first phase transition (i.e., second slope of the free-Ca curve). Lighter blue curves correspond to the Gaussian curves obtained after deconvolution. The bottom line shows the residual.

In addition, NMR results provide further evidence of the liquid–liquid separation occurring in the system. In the range of 5–13 pH, ^31^P NMR spectra of inorganic phosphate solutions show one peak in-between the two chemical shifts of H_2_PO_4_^–^ (ca. 1 ppm) and HPO_4_^2–^ (ca. 6 ppm).^32^ In solution, these two phosphate species quickly interconvert through proton exchange, and the result is an intermediate NMR spectrum. In all measurements performed here, the observed peak shows a chemical shift between 2.42 and 2.43 ppm. Samples analyzed include (1) phosphate buffer, (2) the solution drawn from titration experiments before the first phase transition (i.e., first slope of the free-Ca curve), and (3) the solution drawn from titration experiments after the first phase transition (i.e., second slope of the free-Ca curve). In control runs, a slight broadening of the peak is observed in the samples drawn from titration experiments prior to the first phase transition as compared with the NMR spectrum of the 20 mM phosphate buffer, but this peak remains basically symmetric (Figure 3a,b). However, in the sample drawn from titration after the phase transition (i.e., upon the change in the slope of the free-Ca plot), the peak in the ^31^P spectrum shows a shoulder in the up-field direction (Figure 3c). According to Bewernitz et al.,^30^ this suggests that a fraction of the ions in solution favor H_2_PO_4_^–^ in the H_2_PO_4_^–^–HPO_4_^2–^ exchange to a greater extent than the bulk solution and that this could be caused by the formation of an additional phase in the system. Fitting to Gaussian curves was performed to separate overlapping peaks. The broadened peak can be deconvoluted into two contributions: one main peak that corresponds to the bulk solution as it is similar to the peak observed in the phosphate buffer and a secondary, smaller peak, which can be attributed to the emergent CaP liquid phase. The occurrence of a liquid–liquid phase separation prior to (solid) calcium phosphate formation is critical for bone mineralization, as it allows the infiltration into collagen fibrils of an amorphous, liquid-like mineral precursor to HAp.^3,10^

### 2.2 Effect of Citrate on Prenucleation Events (Formation of PNCs and Liquid–Liquid Separation)

As in the case of the citrate-free experiments, in the presence of citrate (Figure 1b), the measured free calcium first increases linearly with the amount of calcium added and it is lower than the calcium added. Binding of calcium ions to citrate by an ion exchange mechanism can be only inferred at the highest concentration of citrate tested in this study (2 mM); this was identified by a clear shift of the free calcium curve (the dotted line represents the extrapolation of the linear regime of the free calcium curve in Figure 1b). On average, each carboxylate group binds only 0.06–0.07 calcium ions at 1 and 2 mM. This value is quite low compared with the Ca binding by citrate in the absence of phosphate (∼0.12 Ca per COOH^–^).^33^ One the one hand, this confirms that the observed delay in the onset of nucleation in the presence of citrate cannot be caused by a reduction in the effective supersaturation of the system associated with calcium complexation.^34^ On the other hand, it shows that when both carboxylic and phosphate groups are in solution and compete for calcium binding, formation of CaP prenucleation associates becomes more probable than citrate binding to free calcium ions.^34^ Moreover, the presence of citrate also affects the slope of the first linear regime of the free calcium plot, which becomes increasingly flatter with increasing citrate concentration (Figure 1b,c). This slope is related to the stability of Ca–P associates present in solution prior to nucleation (i.e., PNCs or, likely, Posner’s clusters): the flatter the slope the more calcium ions that are bound in solution clusters.^5,33,35^ Carboxylic groups in citrate (−COO^–^) permit interactions of this molecule with calcium ions in solution and Ca–P prenucleation associates. Binding to Ca–P PNCs results in more stable prenucleation species and seems to prevent their aggregation and growth, a required step for the appearance of the HAp precursor liquid phase.^34^ To quantify such an effect, a simplified multiple binding equilibrium was applied for the linear part of the free-calcium concentration curve in the prenucleation regime to determine the standard free energy of cluster formation, Δ*G*_ion_pair_^5^ (see the Supporting Information). It can be concluded that citrate stabilizes the prenucleation species (i.e., more negative values of Δ*G*_ion_pair_ are found in the presence of citrate) (Figure S3).

In addition, this is confirmed by a clear delay in the emergence of the second linear regime with increasing citrate concentration (Figure 1b). Moreover, citrate significantly extends the duration of this second regime (Figure 1b), and the second slope on the free calcium curve is also flatter (Figure 1c), which indicates that more calcium ions are bound in this Ca–P intermediate phase. This shows that citrate is capable of stabilizing the intermediate liquid-like phase formed in our experiments,^34^ delaying its transformation into a solid phase (i.e., ACP, see below). Overall, these two combined effects delay the onset of nucleation of the first solid phase in the system (Figure 1b,d), which provides an alternative explanation for the well-known reported role of citrate as an HAp crystallization inhibitor.^36,37^

TEM observations of the samples drawn from citrate-bearing titration experiments (first arrow in Figure 1b) show what seems to be an earlier stage of the phase-separated structure observed in control runs (see Figure 2a–d). It displays a morphology that closely resembles an emulsion (in the dry state due to quenching with ethanol and subsequent drying), which could well be the solid remnants of a dense liquid phase formed in the system via a spinodal phase separation (Figure 2e–h). The solid residues in this case appear less dense (lighter contrast) and show diffuse edges as compared with the samples drawn in the same regime of the reference experiments (Figure 2a,d). The EDS elemental maps presented in Figure 2f–h were obtained from the analysis of one of these emulsion-like structures. These analyses reveal the presence of Ca, P, and C in this structure. The slightly higher C concentration in the region of interest compared with the C-grid background can be associated with the incorporation of citrate in the liquid intermediate phase.

Based on DLS analysis (see below), it is assumed that these type of structures leads to the formation of solid structures such as the those shown in Figure 2a, most likely by densification via the expulsion of water. ^31^P NMR studies of the samples drawn from citrate-bearing (0.1 mM) titration experiments show that both the buffer sample (Figure S4a) and the solution prior to the first phase transition (Figure S4b) show symmetrical Gaussian distributions of the signal. After the first phase transition (Figure S4c), the evidence of an emergent phase manifests as asymmetric broadening, although in this case such broadening occurs in the downfield direction from the bulk solution peak (i.e., HPO_4_^2–^ direction), which requires an additional Gaussian peak to fit the curve. This again provides an evidence of liquid–liquid separation occurring in the system, although according to these results, the incorporation of citrate in the emergent liquid phase favors HPO_4_^2–^ in the H_2_PO_4_^–^–HPO_4_^2–^ compared to the bulk solution.

### 2.3 Nucleation of Solid Phases

At later stages of the titration runs, the free calcium reaches a point when a first drop is observed in both the citrate-free and citrate-bearing runs. This event coincides with a second change in the transmittance curve, which drops more sharply from this point on and marks the onset of the postnucleation stage (Figure 1). These observations are related to the appearance of the first (metastable) solid phase in the system, ACP. There are many references to different ACPs in the literature, which differ basically in their Ca/P ratios. The most frequent form of ACP, amorphous tricalcium phosphate (ATCP), has an atomic Ca/P ratio of 1.5. Two other “stable” ACPs (ACP1 and ACP2) with a Ca/P ratio of 1.35 have also been reported.^38^ ACPs with lower (1.15) ratios have been also obtained, but they are highly unstable and quickly transform into crystalline phases. Finally, ACP phases with a Ca/P ratio higher than 1.5 have been described but are only formed in the presence of foreign ions.^39^ Solubility data obtained by free Ca measurements in titration experiments correspond to the most soluble phase present in the system. According to our solubility calculations, during the first, short plateau, the solution is just at the equilibrium with respect to ATCP (Table 1), which suggests that the formation of ATCP (the most soluble and therefore the less stable ACP considered here) as the first transient, solid phase in the system is thermodynamically possible. A second drop is seen shortly afterward, preceded by a small but clear plateau in the free calcium plot. This event is related to the transformation of ACTP into a more stable (less soluble) ACP, termed ACP1 (Table 1). Interestingly, this second drop occurs concomitantly with a slight (and temporary) increase in the transmittance of the solution (Figure 1). This is related, according to in situ DLS measurements (as it is discussed below), to the massive aggregation of nanoparticles in solution that clears up most of the reaction media. The final (poorly) crystalline phase is identified as nanocrystalline HAp in all experiments, based on Fourier transform infrared spectroscopy (FTIR) and XRD analysis (see Figures S5 and S6). We did not detect other intermediate crystalline phases, such as octacalcium phosphate (OCP), in our system by any of the analysis performed (i.e., TEM, XRD, or FTIR), and their formation as stable intermediates instead of ATCP or ACP1 would not be consistent with the solubility data presented in Table 1. Therefore, in our system, ACP1 transforms into HAp (both in citrate-free and citrate-bearing experiments) and both phases coexist at the end of the titration runs (immediately after the stabilization of the free-Ca curve, following the second drop described above) (Figure S7).

**Table 1:** Ion Activity Products (IAP) and Saturation Indexes (SI = log (IAP/Ksp)) with Respect to Different Calcium Phosphate Phases

first plateau	KspHAp		KspOCP		KspATCP		KspACP1		KspACP2	
	116.8^66^		96.6^66^		23.9^39^		25.5^39^		28.3^39^	
[citrate] mM	IAP_HAp_	SI_HAp_	IAP_OCP_	SI_OCP_	IAP_ACP0_	SI_ACP0_	IAP_ACP1_	SI_ACP1_	IAP_ACP2_	SI_ACP2_
0	–87.5	29.3	–59.4	37.2	–23.8	0.1	–23.8	1.7	–23.8	4.5
0.1	–87.5	29.3	–59.2	37.4	–23.7	0.2	–23.7	1.8	–23.7	4.6
0.5	–86.8	30.0	–58.6	38	–23.5	0.4	–23.5	2.0	–23.5	4.8
1	–87.6	29.2	–59.3	37.3	–23.8	0.1	–23.8	1.7	–23.8	4.5
2	–86.2	30.6	–58.4	38.2	–23.4	0.5	–23.4	2.1	–23.4	4.9

second plateau	KspHAp		KspOCP		KspACP0		KspACP1		KspACP2	
	116.8^66^		96.6^66^		23.9^39^		25.5^39^		28.3^39^	
[citrate] mM	IAP_HAp_	SI_HAp_	IAP_OCP_	SI_OCP_	IAP_ACP0_	SI_ACP0_	IAP_ACP1_	SI_ACP1_	IAP_ACP2_	SI_ACP2_
0	–93.3	23.5	–63.9	32.7	–25.5	–1.6	–25.5	0.0	–25.5	2.8
0.1	–92.7	24.1	–63.4	33.2	–25.3	–1.4	–25.3	0.2	–25.3	3.0
0.5	–91.8	25.0	–62.6	34.0	–25.0	–1.1	–25.0	0.5	–25.0	3.3
1	–92.9	23.9	–63.6	33.0	–25.4	–1.5	–25.4	0.1	–25.4	2.9
2	–91.6	25.2	–62.7	33.9	–25.0	–1.1	–25.0	0.5	–25.0	3.3

### 2.4 Morphology of HAp Precipitates

**Figure 4:**
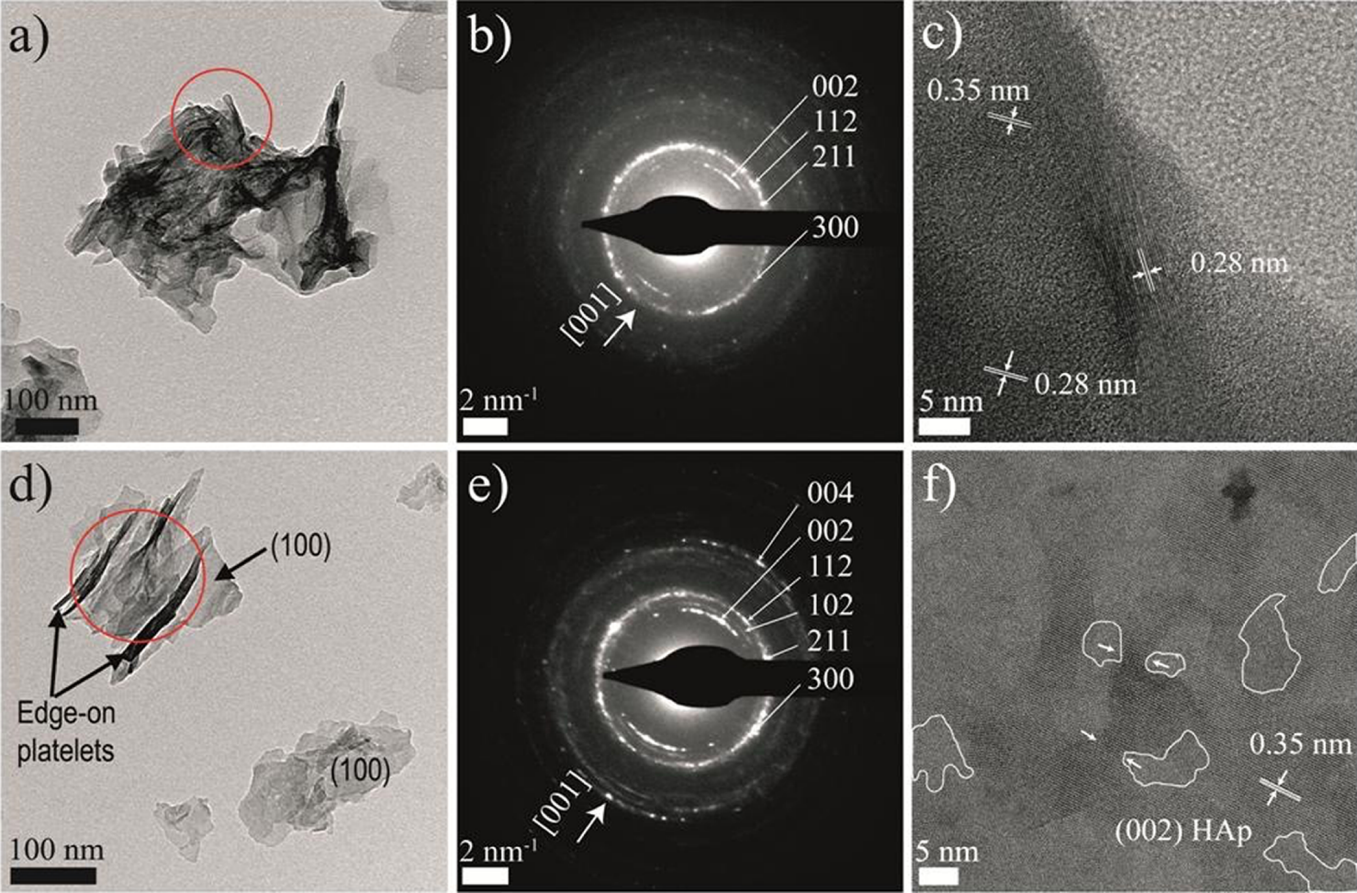
TEM images of the aggregates of HAp particles obtained after the titration experiments performed at pH 8: (a–c) 0 and (d–f) 0.5 mM citrate. The SAED pattern of the aggregate (red circled area) in (a) and (d) (shown in (b) and (e), respectively; *hkl* indexing of HAp—for space group *P*6_3_/*m*—is indicated) shows that the nanoparticles have a certain degree of preferred orientation along the *c* axis. HRTEM imaging shows misorientation among different domains in (c). Small amorphous domains in between crystalline areas with HAp lattice spacing are seen in (c) and (f). Necklike structures connecting crystalline domains are marked by white arrows in (f).

With and without citrate, HAp precipitates consist of aggregates of nanosized particles with irregular morphologies (Figures 4 and S8). Low-resolution TEM images show these structures as the typical HAp aggregates of sheetlike nanoparticles ca. 5–15 nm in thickness and ca. 50–300 nm in length, in many cases showing a ribbonlike morphology due to bending (best observed in nanoplates oriented edge-on). No significant differences in the morphology or size of the aggregates can be inferred from high-resolution field emission scanning electron microscopy (FESEM) (Figure S8) or the TEM (Figure 4) images of the precipitates formed in the absence and in the presence of citrate (second arrow in Figure 1b), except for a slight reduction in the thickness of the nanoplates in the presence of 0.5 mM citrate (from 12.2 ± 2.1 nm, *N* = 22, in control runs to 6.7 ± 2.2 nm, *N* = 18, in citrate experiments).

It is commonly accepted that HAp can be found in two crystallographic forms, the hexagonal phase having the *P*6_3_/*m* space group and the monoclinic phase, with the space group *P*2_1_/*b*. Nevertheless, it has been reported that HAp in the polycrystalline or thin platelet form as it occurs in bone or in our study may be best represented by a second monoclinic, energetically favorable *P*2_1_ phase.^40^ However, concerning the interpretation of our experimental XRPD diagrams, we adopted the hexagonal symmetry, according to (JCPDS 9-432), for the sake of practice and simplicity. Note that the indexing of the hexagonal cell can readily be transformed into the monoclinic (pseudohexagonal) indexing, considering the following zone axis relationships: [0001]//[001], [1100]//[110], and [2110]//[100].^41^

The intense diffraction arcs at *d*-spacings of 0.35 and 0.17 nm corresponding to the 002 and 004 Bragg reflections, respectively (Figure 4b,e), indicate a preferred orientation along the *c* axis of the HAp nanoplates in the aggregates shown in Figure 4a,d, further corroborated by the fact that the intensity of the 002 reflection in the XRD pattern is increased relative to that of the 121 reflection (*I*_002_/*I*_121_ = 0.38—PDF pattern—vs *I*_002_/*I*_121_ = 0.74—HAp in this study, control run). TEM-SAED analysis of isolated plate-like nanoparticles a few nm thick with (100) as the most prominent face (Figure S9), demonstrating that the fiber-like particles observed in the TEM (e.g., Figure 4d) are in fact edge-on platelets. However, the angular spreading of the 002 reflections shows that the nanocrystallites are not in a perfect crystallographic register. The degree of the orientation of the HAp crystals measured for control samples is in the range of 35.4° ± 1.2° (based on the angular spread of the 002 reflection in SAED images). TEM analysis of mature cortical bone yields values close to 36°,^42^ which may increase to higher values in less mature bone and different types of bone and local orientations.^43^ Similarly, the appearance of 003, 112, and 211 reflections corresponding to nontautozonal planes in the SAED (see inset in Figure 4c), shows that a nonperfect orientation along the *a*–*b* plane exists, which is systematically observed in bone HAp.^44^ Altogether, these structural features are defining characteristics of HAp in bone^44^ and indicate that, contrary to current knowledge,^45^ they can be achieved without the presence of any organic molecules or the templating action of collagen. The SAED pattern of precipitates formed in the presence of citrate shows an angular spreading of the 002 reflection of 40.6° ± 0.2°.

There has been significant research on the structure and organization of HAp platelets formed in bone and in vitro. In addition, the morphology and habit of HAp crystals, both from pure solution and in the presence of substrates and/or foreign substances (including citrate), have been systematically studied by Aquilano and co-workers in the last decade.^46,47,48^ HAp platelets formed both in vitro and in vivo grow elongated parallel to the *c* axis, with (100) as the most developed face, as shown, for example, by Moradian-Oldak and co-workers.^49^ Different hypotheses have been proposed to explain the formation of HAp with platelet morphology, a phase that, if one assumes it is hexagonal (space group P63/m), should not develop that shape.^24^ Moradian-Oldak and co-workers^49^ argue that such a morphology is the result of the formation of HAp from a crystalline precursor having that shape (i.e., OCP). This is not congruent with our results or with those by other researchers,^49^ where ACP directly transforms into HAp, without the participation of any other crystalline phase. Another hypothesis proposed is the preferential adsorption of citrate on the (100) faces.^24^ However, this is not consistent with our results showing that the same morphology is observed in the absence of citrate. Recent work shows that HAp platelets in bone appear to be formed by a nonclassical mechanism implying lateral aggregation of fibers developed after an amorphous precursor.^50^ In line with this work, our observations suggest that the most probable mechanism for the formation of these plates is a nonclassical process of aggregation along [010] of fibers or, more exactly, very thin and elongated plates, which have formed from ACP and grow preferably along the *c* direction.^51^

HRTEM images show that each of these nanoparticles formed in the absence and presence of citrate is composed of small domains with no lattice fringes in between crystalline areas. Sample tilting of these particles was used to verify that these regions are indeed amorphous areas (ACP), while in the case of samples taken from control runs, different orientations of the same planes were observed (see Figure 4b); in citrate-bearing samples, crystalline regions mostly present a single crystal orientation (Figure 4c). The HRTEM images and their fast Fourier transforms are consistent with the crystalline areas having the crystallographic structure of HAp (e.g., *d*-spacing of 0.35 nm, corresponding to the (002) planes of HAp). Crystalline domains are connected by necklike bridges that suggest that HAp platelets have grown by a nanoparticle-mediated nonclassical mechanism; such grain boundaries could have formed by a mechanism similar to the coalescence-driven crystal growth mechanism, recently proposed for bismuth crystal growth and rearrangement.^52^ Interestingly, a recent report shows that HAp can grow along a preferred *c* axis orientation via the anisotropic epitaxial crystallization of ACP onto HAp, irrespective of the crystallographic orientation of the HAp substrate.^49^ Molecular dynamics modeling suggests that this is due to the fact that the (001) planes of HAp are the ones with the lowest surface energy, an effect that energetically favors the incorporation of growth units into the crystalline structure of HAp along the [001] direction. These results may help to explain why in our experiments (and possibly in bone) HAp nanoplates are elongated along [001] and why a preferred *c* axis orientation of the HAp nanoparticles formed after ACP is observed in the absence of any template. They could attach laterally in the *b* axis direction, giving the plate-like morphology that are observed in our experiments and in bone samples. In addition, the sheetlike particles are very poorly crystalline and contain a huge number of defects, including amorphous areas, as shown by our HRTEM images. These characteristics would allow them to bend/twist in the *c* direction, which is ultimately reflected in the final curvature of the platelets seen in our TEM images and in bones.^50^

However, a key question arises: why do they not grow or attach in other directions, finally forming a structure in hexagonal prisms, as it would be expected if we consider HAp having a hexagonal crystal structure? This could be explained considering that the platelike HAp nanocrystals that make up the bones or the precipitates formed in our experiments (via an amorphous precursor) are not hexagonal but monoclinic, with a pseudohexagonal cell unit in which the parameter *b* = 2*a*. This helps explaining their anisotropic growth, as it is discussed in this work. This was already proposed by Elliott in 1971^53^ and later demonstrated by XRD,^54^ and since then, there are quite a few references on the monoclinic structure of HAp,^55,56^ including a study on its formation at relatively low *T*.^57^

Detailed investigations of the aggregation processes during HAp formation are beyond the scope of this report, but the combined FESEM and TEM observations reported here suggest that sheetlike HAp crystals are formed by coalescence of crystalline and/or amorphous nanoparticles. Furthermore, this agrees with recent in situ liquid-phase TEM showing that calcium phosphate mineralizes by particle attachment.^58^

### 2.5 ζ-Potential and Evolution of Particle Size

**Figure 5:**
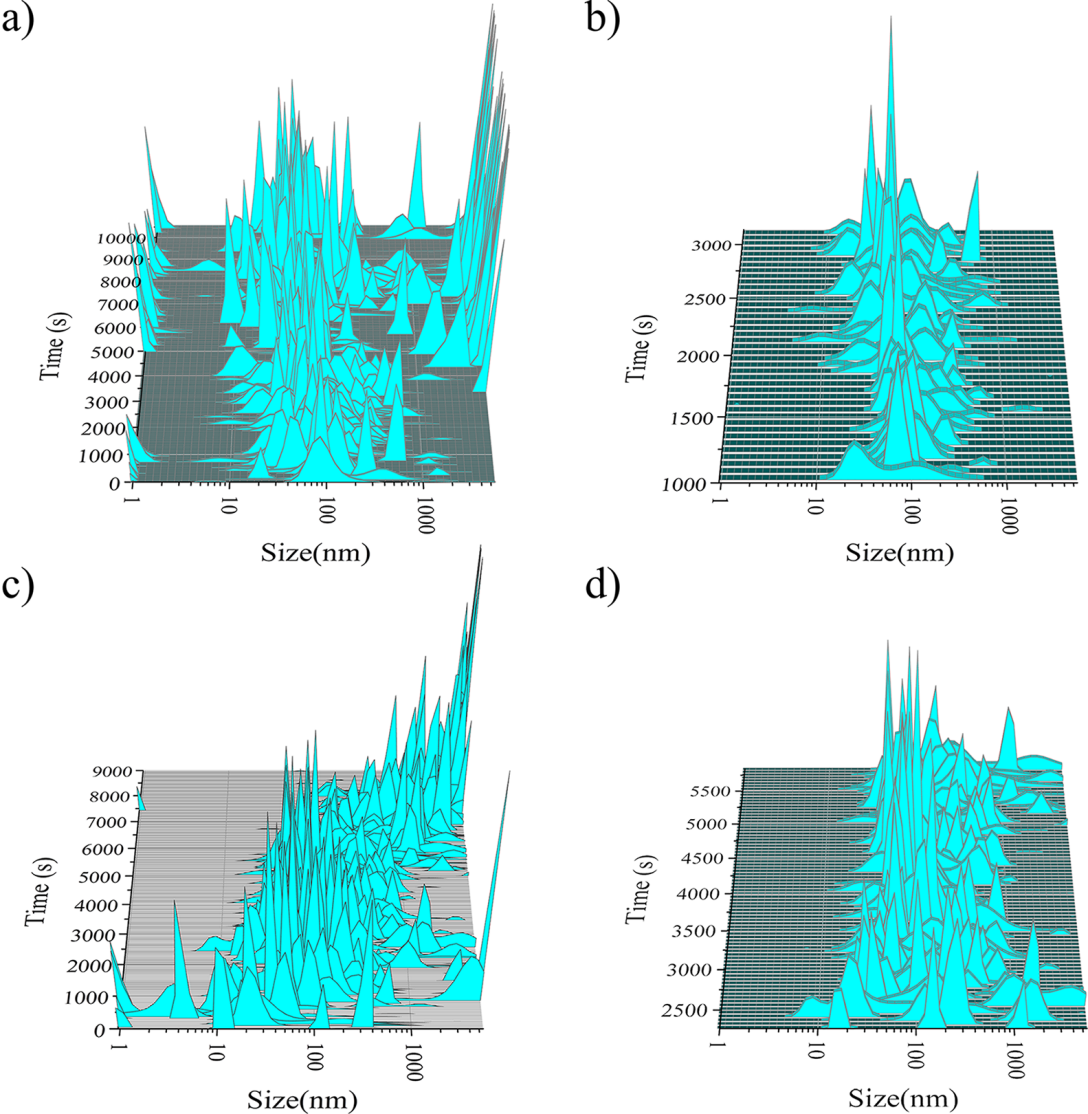
Results of in situ DLS measurements performed during titration experiments at pH 8. (a) Control run (0 mM citrate), showing the abrupt appearance of micron-size particles at ca. 4000 s; (b) zoomed image of (a) in the 1–3000 s region; (c) 0.5 mM citrate solution; and (d) zoomed image of (c) in the 2250–5750 s region, showing the wider size distribution starting from ca. 2500 s.

The regimes described above, based on the evolution of the free calcium, can be distinctly traced by in situ DLS (Figure 5). In control runs, species with sizes ranging from ∼30 to ∼100 nm are detected already during the early stages of the experiments (i.e., before the first change in the slope of the free calcium plot). These species are interpreted to be the aggregates of prenucleation clusters. Note that a similar size range (20–200 nm) has been reported for aggregates of PNCs in CaCO_3_.^59^ After the first phase transition seen in the control runs at ca. 1200 s (marked by a change in the slopes of the transmittance and free calcium concentration curves), a slight increase in size dispersion is observed (sizes ranging from 10 up to 900 nm). This is consistent with the occurrence of a dense liquid phase within the system because of the liquid–liquid phase separation described above. After ca. 3000 s (concurring with the first drop in the free Ca), two different populations are observed: one with particle sizes between 10 and 200 nm that could correspond to both ACP and/or HAp individual nanoparticles, and a second set of particles with sizes mostly above 6 μm, which correspond to the large aggregates of these nanoparticles seen in the TEM and FESEM observations of the final precipitates.

Interestingly, in the case of citrate, an initial (*t* < 2000 s) formation of a population of particles with a diameter below 10 nm is observed, which quickly increases to 100 nm. These species are 1 order of magnitude larger than Posner’s clusters and are more likely small aggregates of prenucleation clusters, which seem stable with respect to ions in solution, because otherwise they would appear as fluctuations in the solution and would not tend to grow with increasing calcium concentration in the reaction media.^29^ These particles are not detected in control runs, most likely because they quickly aggregate and grow in the absence of citrate, while citrate—temporarily—stabilizes them, in agreement with our previous results.^60^ After 2000 s, species show a broader particle size distribution, spanning from 10 nm up to >1000 nm. Again, this observation agrees with a liquid–liquid phase separation taking place in the system. With an increase in the calcium concentration, a slight tendency to decrease in the size and polydispersity of this phase is observed, possibly because of a densification process. Finally, from ca. 6000 s and coinciding with the second drop in the free calcium concentration plot, a very rapid increase in the particle size up to sizes above 6 μm is observed. Interestingly, the individual nanoparticles of sizes between 10 and 200 nm seen in control runs are not detected here. The ACP and/or HAp nanoparticles formed in the presence of citrate seem to have a strong tendency to aggregate, leading to final micrometric particles.

Zeta potential measurements conducted on samples drawn in the final plateau of the experiments depict a slightly negative zeta potential of −5.4 mV for citrate-free HAp. In solution, HAp particles present a highly mobile layer of ionic species on the surface, whose composition changes readily with the chemistry of the solution.^61^ This, together with the low solubility of HAp and its slow growth rate, results in a complex charge distribution on the crystal surface and in the double layer of ions in the solution in contact with the crystal surface. As a result, the chemistry of the solution from which HAp is precipitated has a marked effect on the ζ-potential of the particles that could explain the wide variety of values found in the literature.^61^ In the presence of citrate, negative zeta potential values ranging between −21.8 and −31.9 mV were measured for precipitated HAp and/or APC1. It is straightforward that these more negative values are related to the adsorption or incorporation of citrate species on Ca–P phases. The presence of adsorbed and/or incorporated citrate on the final precipitated phase (HAp) is further corroborated by our FTIR (Figure S5) and STEM-EDS analysis (Figure S7). Further studies are necessary to confirm or exclude the possible incorporation of citrate into HAp; however, it is clear from our measurements that citrate is at least adsorbed on the surface of HAp nanoparticles. Incorporation and adsorption of citrate into ACP have been previously demonstrated.^21,62^ Citrate-bearing ACP has been shown to carry a negative surface charge, which would, in principle, hamper ACP nanoparticle aggregation; however, aggregation could occur instantaneously upon the release of adsorbed citrate by ionic exchange with the excess phosphate in the reaction media.^21^ Upon citrate release and subsequent aggregation, contacting ACP nanoparticles could crystallize by a coalescence mechanism,^52^ as indicated above, which could be driven by the decrease in surface energy related to the increase in size. Alternatively, the release of the adsorbed citrate would decrease the stability of ACP that would subsequently dissolve, releasing potentially incorporated citrate, and recrystallize as HAp. Indeed, previous studies have shown that citrate desorption from ACP surfaces prompt HAp crystallization.^21^ Further studies would be needed to unravel the actual mechanism for such transformations, but it is clear from our HRTEM results showing amorphous domains within HAp crystals that full dissolution or transformation of ACP is not achieved during our experiments.

## 3 Conclusions and Outlook

In conclusion, we show how a kinetically induced dense liquid precursor is formed prior to HAp crystallization and ACP nucleation during calcium phosphate formation, which is stabilized in the presence of citrate. Our findings provide a mechanism that explains earlier claims that citrate improves collagen-mediated mineralization by calcium phosphate, a role that has been previously assigned to macromolecules (NCPs). Moreover, we have demonstrated that citrate is also able to stabilize calcium phosphate prenucleation species and consequently delay the formation of any liquid or solid phase. The presence of citrate most likely prevents their aggregation that is a required step in the formation of the CaP liquid precursor phase in the system. Moreover, while previous work relates the inhibition of HAp crystallization by citrate with the fact that surface-adsorbed citrate prevents ACP to HAp transformation,^21,62^ or with the ability of citrate to complex Ca in solution,^25^ we show that by stabilizing early CaP precursors (prenucleation species and liquid precursor phase) citrate is able to delay solid calcium phosphate nucleation extensively. Furthermore, since HAp nanoplatelets are observed both in the absence and in the presence of citrate, models that attribute to citrate an effect as a habit modifier due to the adsorption on (100)^24^ should be revised. It is worth mentioning that the stabilization of this Ca–P liquid phase by citrate stands as a promising strategy for remineralization purposes (e.g., repairing lesions in dentistry) due to its ability to infiltrating cavities.

It is also remarkable that the formation of HAp after ACP in the absence of any template results in a few nanometer thick HAp nanoplates with (100) as the most prominent faces that self-assemble with a preferred *c* axis orientation. These results challenge the current model for bone mineralization assuming that the morphology and size (and thickness) of HAp nanocrystals in bone as well as its *c* axis preferred orientation is basically due the space-constraints within collagen fibrils and to a templating effect of collagen. The nanoplate morphology of HAp crystals appear to be an intrinsic feature of HAp formed from an amorphous precursor, likely because it is a monoclinic phase, which provides a possible explanation for the evolutionary selection of HAp as a bone mineral. This indeed is not accidental for several reasons (many of them already known, such as its hardness, low solubility, or the fact that it represents a Ca and P reservoir), among which it may be critical that, as shown by our results, it tends to form ultrathin nanoplatelets with growth and aggregate along the *c* axis without a template effect. Note, however, that the *c* axis orientation of HAp parallel to the long axis of collagen fibrils in bone is likely imprinted due to a template effect associated with the initial crystallization of HAp (after ACP) at nucleation sites on collagen.^45^ But according to our results, further development of HAp nanoplatelets with a preferred *c* axis orientation can take place without a collagen template effect further apart from the nucleation sites. There is experimental evidence showing that a confining geometry, as that existing between collagen fibrils, can induce the growth of HAp nanoplates after ACP, with their *c* axis preferentially aligned parallel to the longest axis of the confining capillaries.^63^ The latter gives further support to our claim that the intrinsic crystallization and growth nanofeatures of HAp formed after ACP might be at the root of the specific (nano)features of HAp in bone. HAp platelets formed by nanoparticles (both crystalline and amorphous) will offer greater mechanical resistance as (i) the probability of having critical flaws will be low due to the small size of the building units^64^ and (ii) the remaining ACP in-between HAp nanoparticles, seen in our HRTEM images and in bone HAp,^64^ will act as an additional reinforcement of this crystalline–amorphous hybrid material preventing the propagation of fractures, as it has been shown in other biominerals. This is another key observation of our work that contributes to the understanding of the mechanical characteristics of bone. Finally, the ability to form amorphous, liquid-like mineral precursor to the final crystalline phase is critical for bone mineralization, as it allows the infiltration into collagen fibrils, and it is facilitated/promoted by citrate molecules as shown by our results. This is of particular relevance given the size-exclusion characteristics of collagen, which prevent molecules larger than 40 kDa to penetrate into collagen fibrils.^65^ Thus, liquid-like HAp precursor phases with large NCPs adsorbed would not be able to mineralize collagen, while drops of the citrate-stabilized, amorphous Ca–P precursor will freely access the intrafibrillar space in collagen. The function of large NCPs such as fetuin that are not able to enter the fibrils is related to the inhibition of apatite growth everywhere else except inside the fibril.^66^ Altogether, this fundamental study reveals previously unknown key aspects of the formation of HAp and the effects of citrate on the early processes involved in its crystallization, thus contributing to improve our understanding of HAp biomineralization.

## 4 Experimental Section

### 4.1 In Situ Ca–P Precipitation Experiments

Potentiometric calcium titration experiments were performed at pH 8.00, kept constant by continuous NaOH addition, using a Titrino 905 manufactured by Methrom. ACS grade reactants (>99% purity) were purchased from Sigma-Aldrich. A 20 mM calcium chloride solution was added to a 20 mM phosphate solution at three different rates: 0.1, 0.5, and 2.4 mL min^–1^. This was done for a range of citrate concentrations in the phosphate buffer solution (0–2 mM). Free calcium concentration was continuously measured using an ion-selective electrode, ISE (Mettler-Toledo, DX240-Ca), calibrated for each experiment by titration of a 10 mM CaCl_2_ into a NaCl solution of the same ionic strength of the corresponding phosphate-citrate-bearing solution. The pH was monitored using a glass electrode (Metrohm), which was simultaneously used as the reference for the Ca ISE. Solution conductivity and transmittance were also continuously recorded during the titration experiments.

Particle size evolution was in situ and continuously monitored by DLS during the precipitation experiments, using the controlled reference heterodyne method as in Ruiz-Agudo et al.^33^ We used a Microtrac NANO-flex particle size analyzer equipped with a diode laser (λ = 780 nm, 5 mW) and a 1 m long flexible measuring probe (diameter = 8 mm) with sapphire window as the sample interface, with an acquisition time per run of 45 s and waiting time between individual measurements of 20 s. Size distributions were calculated using the Microtrac FLEX software (v.11.1.0.1). Finally, aliquots from the precipitation solution were collected upon nucleation in control and 2 mM citrate titration experiments and immediately transferred into a Microtrac Stabino equipment for **ζ**-potential measurements using the streaming potential method.

### 4.2 Ex Situ Analysis of Phase Evolution

For further characterization of the early stages of calcium phosphate precipitation in the presence and absence of citrate, samples were collected from the reaction media at different reaction times, quenched in ethanol, transferred onto carbon-coated copper grids, and observed using a 30 μm objective aperture in a FEI TEM Titan working at 300 kV. SAED patterns were collected using a 10 μm aperture, which allowed the collection of diffraction data from a circular area ∼0.2 μm in diameter. Elemental compositional maps were obtained in the STEM mode using a Super X EDS detector (FEI), formed by four SSD detectors with no window surrounding the sample. STEM images in the FEI Titan TEM of the areas analyzed by EDS were collected with a high-angle annular dark field detector. At the end of each titration experiment, the solution was separated from the reaction media by filtration (Nucleopore, *Ø* = 200 nm), and the solids collected were studied by X-ray diffraction (PANalytical X’Pert Pro X-ray, Cu Kα radiation, λ = 1.5405 A°, 3°–50° 2θ range, scanning rate of 0.11° 2θ s^–1^), FTIR (ATRproONE-FTIR, Jasco Model 6600, frequency range of 400–4000 cm^–1^, resolution of 2 cm^–1^, and 100 accumulations), FESEM (Zeiss SUPRA40VP), and TEM (FEI Titan, 300 kV).

### 4.3 ^31^P NMR Measurements

All NMR experiments were conducted on a Bruker Avance DRX 500 MHz vertical bore system using a *xyz* gradient TXI probe with a 1H and 2H interior coil, 13C and 15N exterior coil, and *xyz* gradients. The analyzed samples were prepared during titration experiments such as those described above except using deuterated water in the preparation of the starting solutions. All experiments were conducted at 25 °C. Data were processed using the MestReNova software for the deconvolution of overlapping spectral peaks.

### 4.4 Isothermal Pseudotitration Calorimetry

It was conducted with the aim of corroborating the occurrence of a phase transition in the system when the formation of a new phase could not be directly detected by other means. The analysis was performed using a punctuated titration method to measure the enthalpy change upon nucleation of the emergent phase. The reactions were performed in a fully silvered Dewar flask of a PARR 6755 solution calorimeter equipped with a PARR 6772 high-precision thermometer (resolution: 10^–4^ from 0 to 70 °C). The energy equivalent of the calorimeter was calibrated several times during the experimental series. To simulate the calcium phosphate precipitation experiments, the Dewar flask and the glass cell (sealed with a detachable Teflon dish) were filled with variable volumes of phosphate (in the absence and presence of citrate) and calcium chloride solutions, respectively. As soon as the thermal equilibrium was achieved in the calorimeter before the reaction, the glass cell was opened into the Dewar flask and the solutions were mixed (95 mL total volume). During the reaction, the cell was continuously rotated by an external electric motor until the end of the experiment.

## Supporting Information Available

Figures S1–S11; application of a multiple binding equilibrium model for estimation of the standard free energy of cluster formation; and XRD and FTIR identification of precipitates (PDF)
