# Supplementary material for: Citrate Stabilizes Hydroxylapatite Precursors: Implications for Bone Mineralization
Source: ACS Biomater Sci Eng. 2021 May 11;7(6):2346–57. doi: 10.1021/acsbiomaterials.1c00196 (PMC8479724; doi:10.1021/acsbiomaterials.1c00196)
Supplement: Supplementary file 1 — ab1c00196_si_001.pdf [file ab1c00196_si_001.pdf]

# Supporting Information

## Citrate stabilizes hydroxylapatite precursors: implications for bone mineralization

*Encarnacion Ruiz-Agudo[1](*), Cristina Ruiz-Agudo[2], Fulvio di Lorenzo[1,3], Pedro Alvarez-Lloret[4], Aurelia Ibañez-Velasco[1], Carlos Rodriguez-Navarro[1]*

*[1] Department of Mineralogy and Petrology, University of Granada, Fuentenueva s/n, 18071 Granada, Spain*
*[2]Physical Chemistry, Department of Chemistry, University of Konstanz, Universitätsstraße 10, 78457 Konstanz, Germany*
*[3]Institute of Geological Sciences, University of Bern, CH-3012, Switzerland.*
*[4]Department of Geology. University of Oviedo. C/ Jesús Arias de Velasco s/n. 33005 Oviedo, Spain*

12 Pages, 11 Figures

## 1. Supplementary Figures

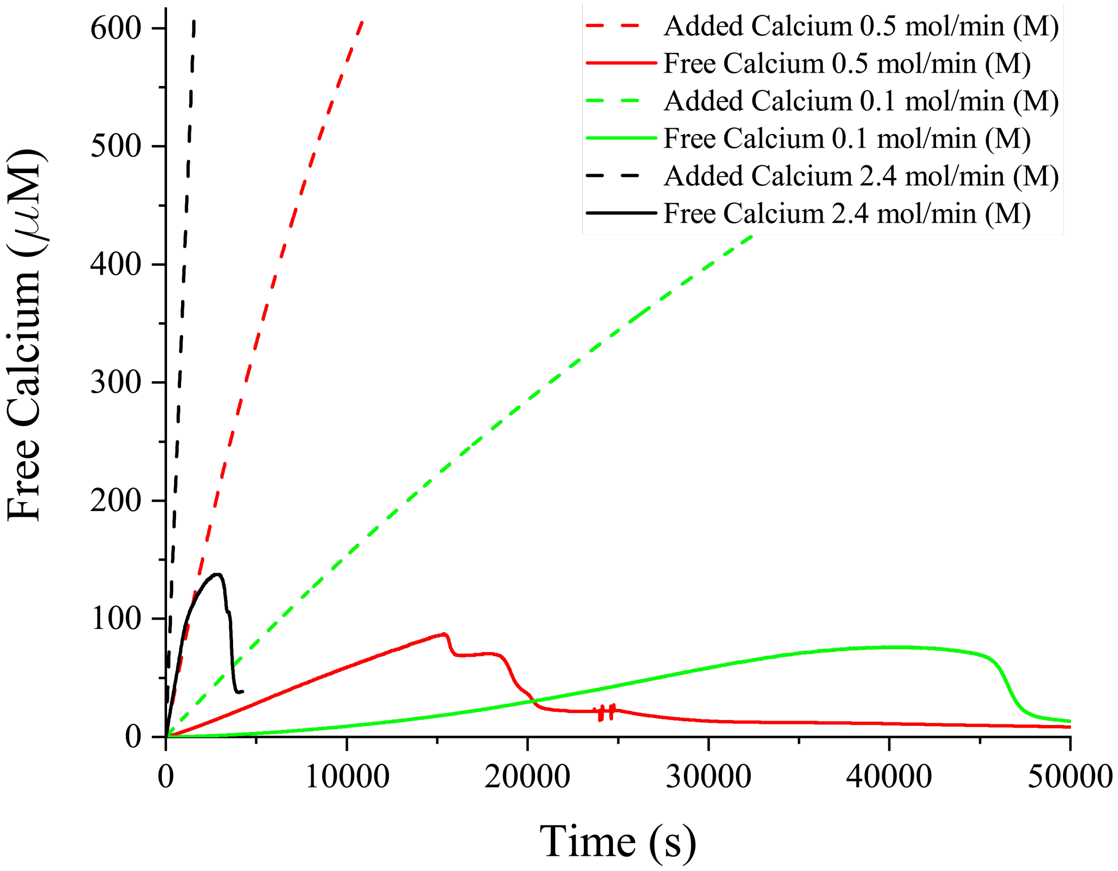


**Figure S1.** Free calcium development curves in control titration experiments performed at different calcium addition rates.

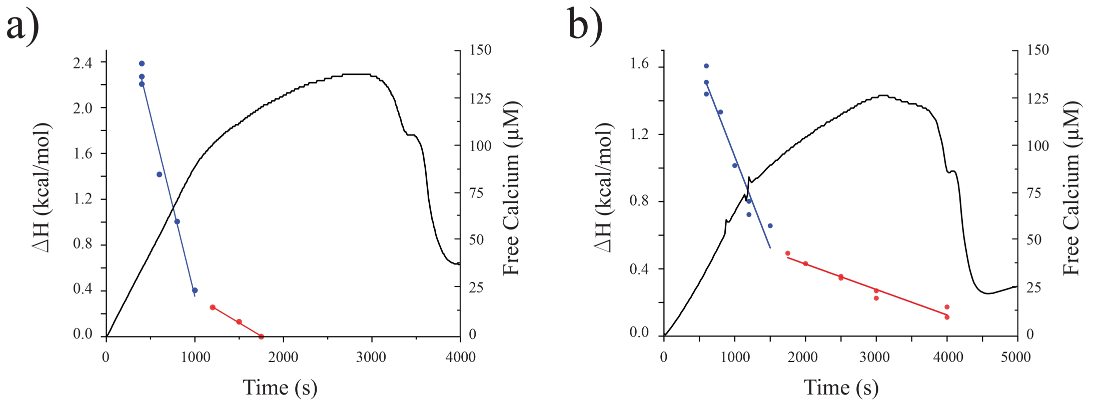


**Figure S2.** The enthalpy of reaction during the titration of $CaCl_2(aq)$ into 20 mM phosphate solution at pH 8 in the presence of a) 0 and b) 0.5 mM citrate. The data show a discontinuity at a) ca. 1200 s and b) ca. 1750 s, pointing to the occurrence of a

phase transition in the system. The black curves represent the free calcium evolution during titration experiments (secondary axes).

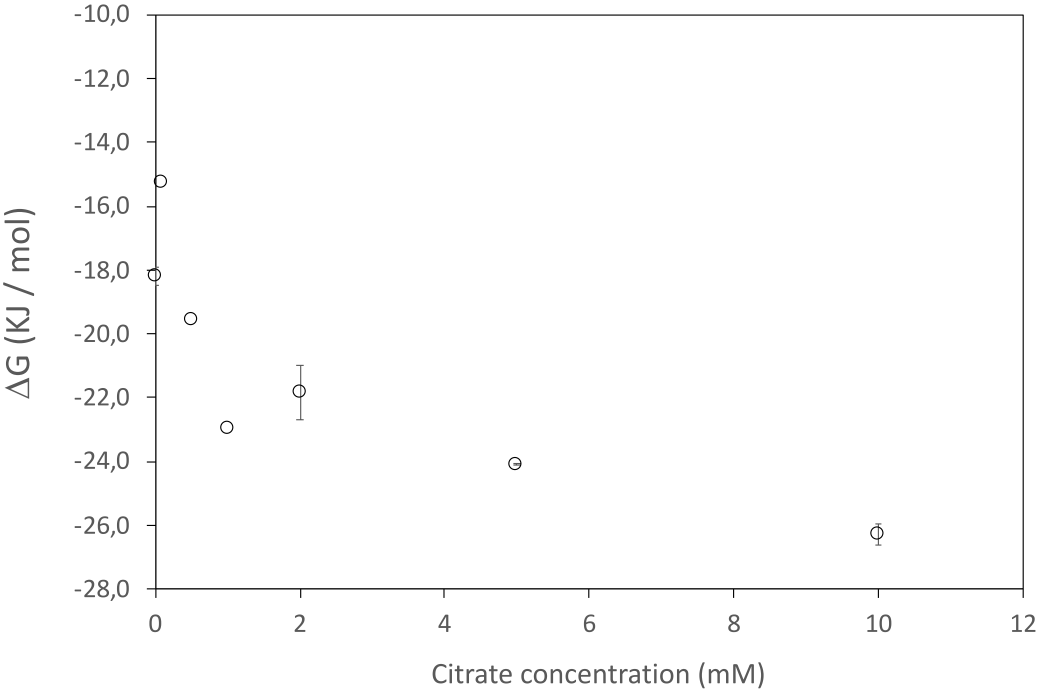


**Figure S3.** Gibbs standard energy for the formation of calcium/phosphate ion pairs in calcium phosphate clusters, as a function of citrate concentration. The data show a decreasing trend, indicating that the presence of citrate increases the stability of calcium phosphate clusters.

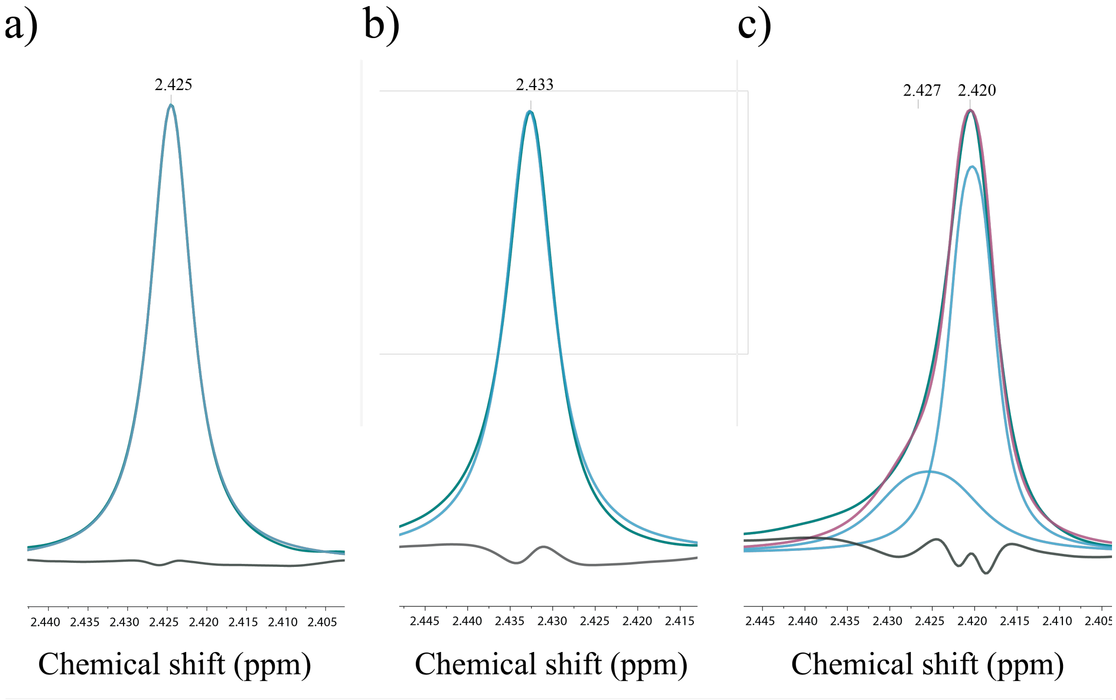


**Figure S4.** 1-D $^{31}P$ NMR spectrum of a) 20 mM phosphate buffer (with citrate added), pH 8.0; b) solution sample drawn from titration experiments before the first phase transition (i.e. first slope of the free-Ca curve) and c) sample drawn from titration experiments after the first phase transition (i.e. second slope of the free-Ca curve).

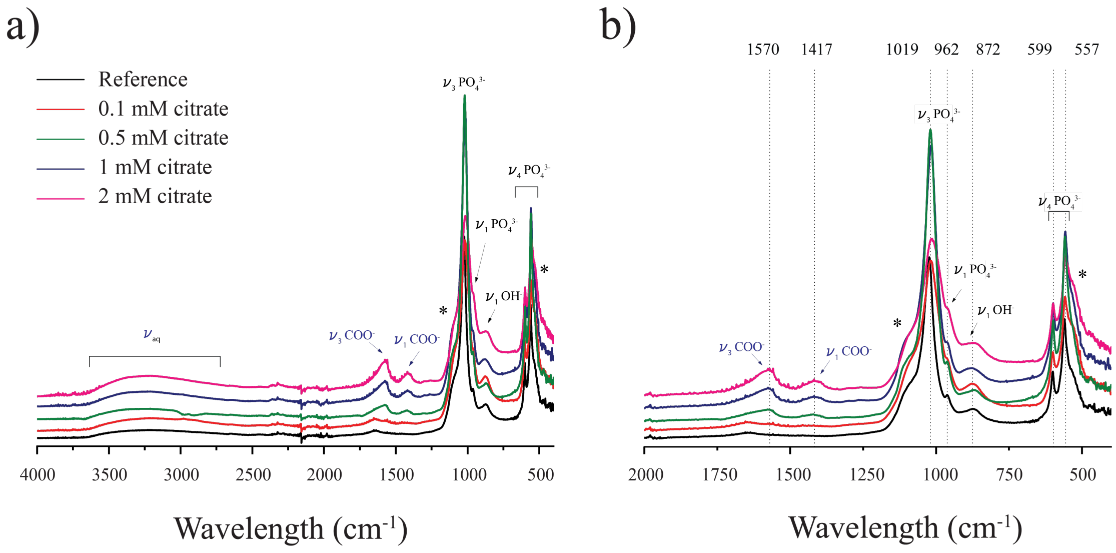


**Figure S5.** IR spectra of precipitates formed in titration experiments performed at pH 8 in the presence of different concentrations of citrate.

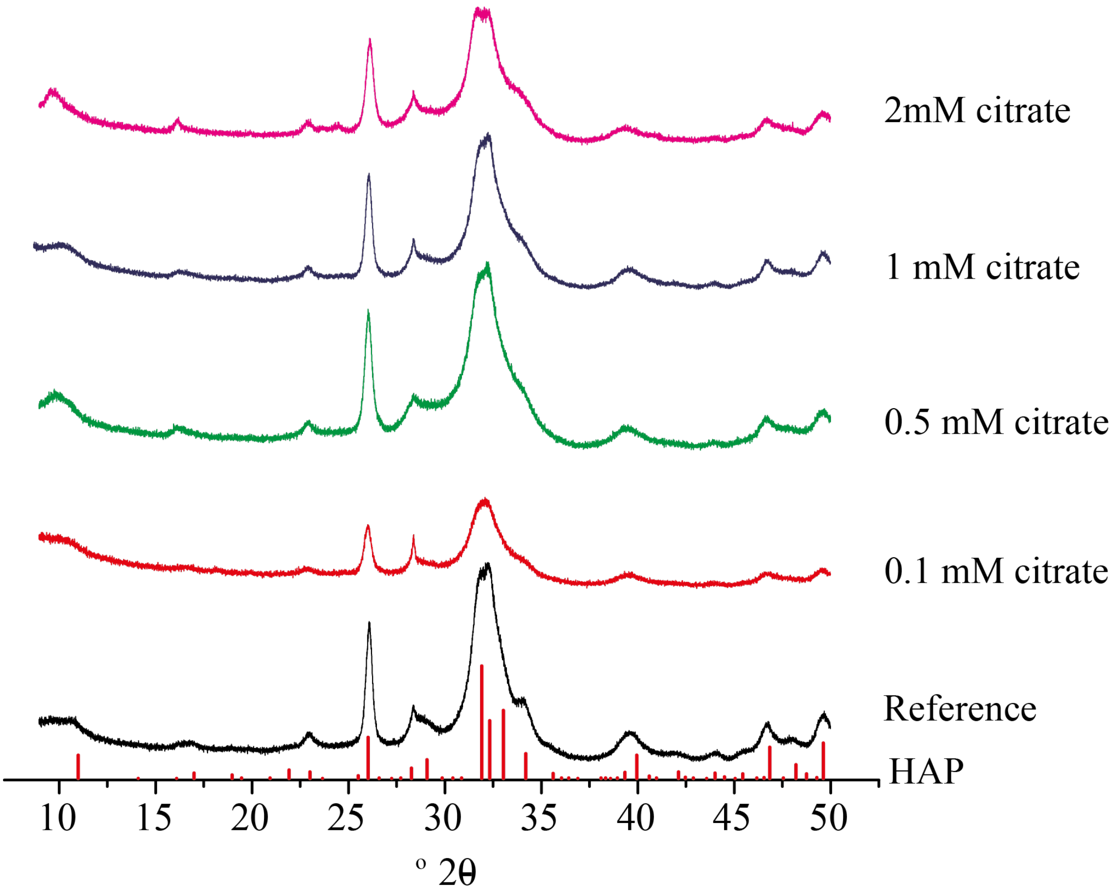


**Figure S6.** XRD pattern of precipitates formed in titration experiments performed at pH 8 in the presence of different concentrations of citrate.

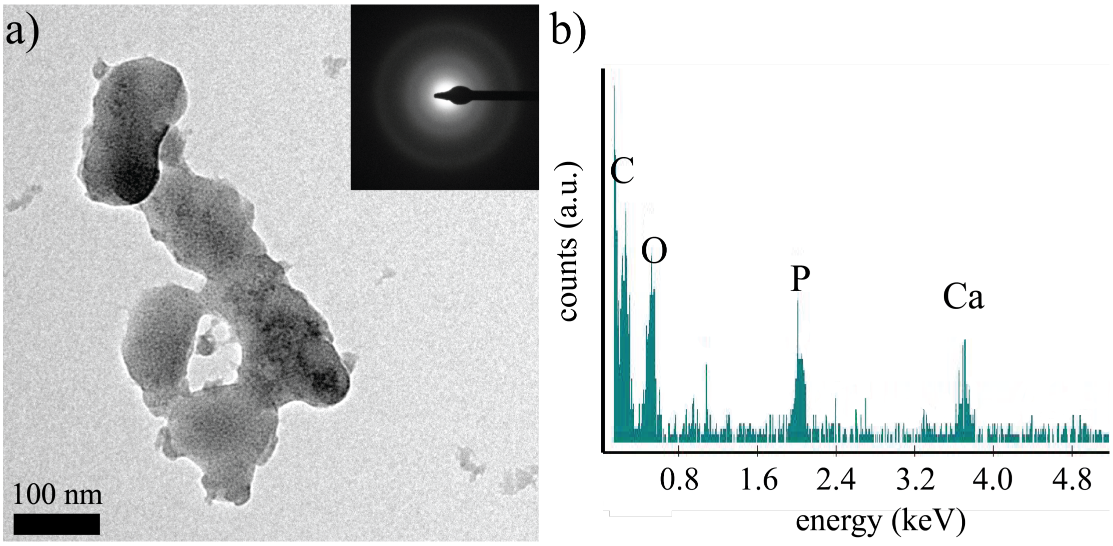

**Figure S7.** a) TEM micrographs of scarce amorphous CaP particles found at the end of the experiments carried out in the presence of 0.5 mM citrate. SAED pattern in inset. b) EDS analysis of the particles in a).

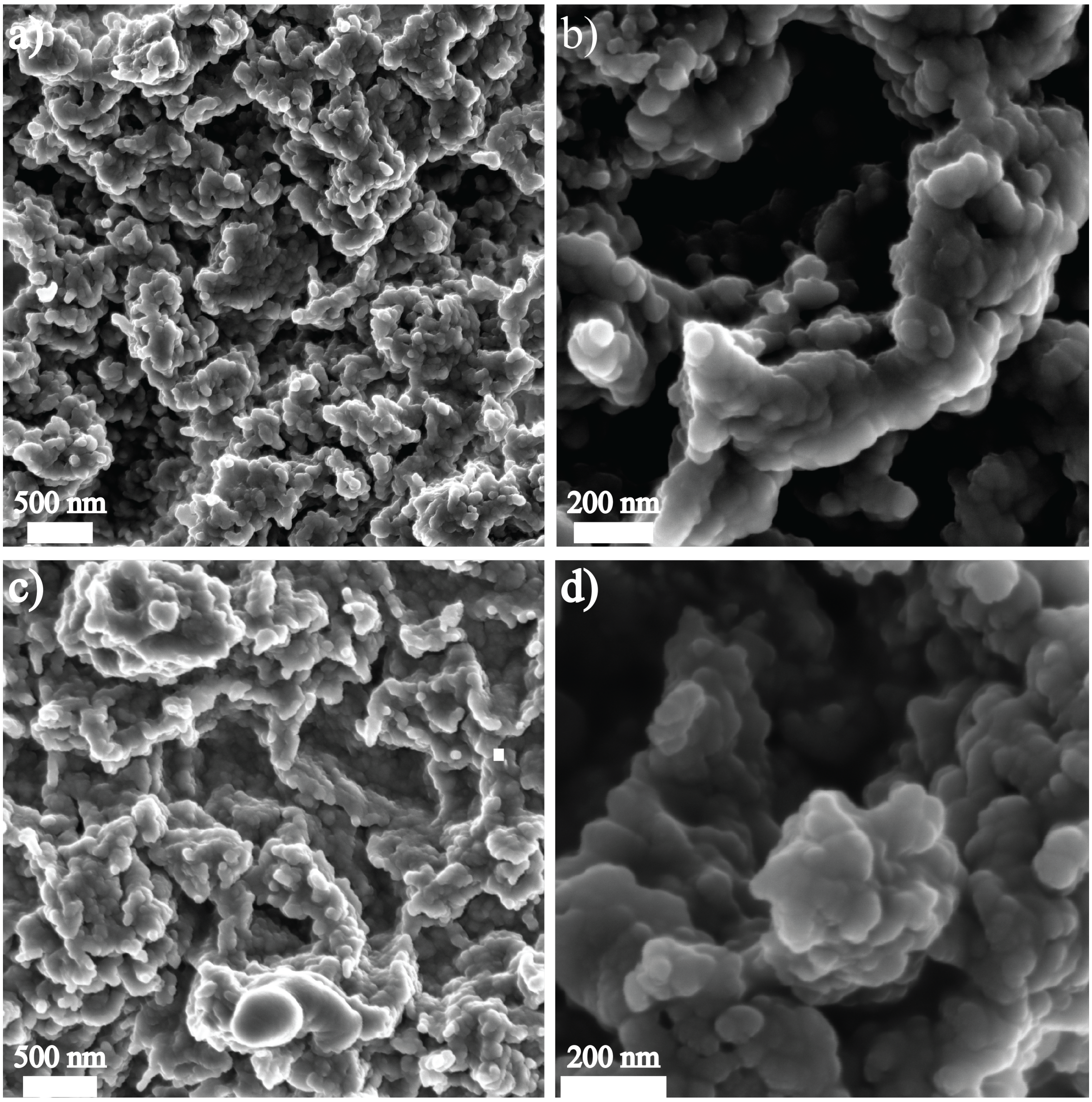


**Figure S8.** FESEM images of calcium phosphate particles (HAp, according to our XRD and FTIR analysis) obtained after the titration experiments performed at pH 8: a,b) 0 (second black arrow in in Figure 2) and c, d) 0.5 mM citrate (second green arrow in Figure 2).

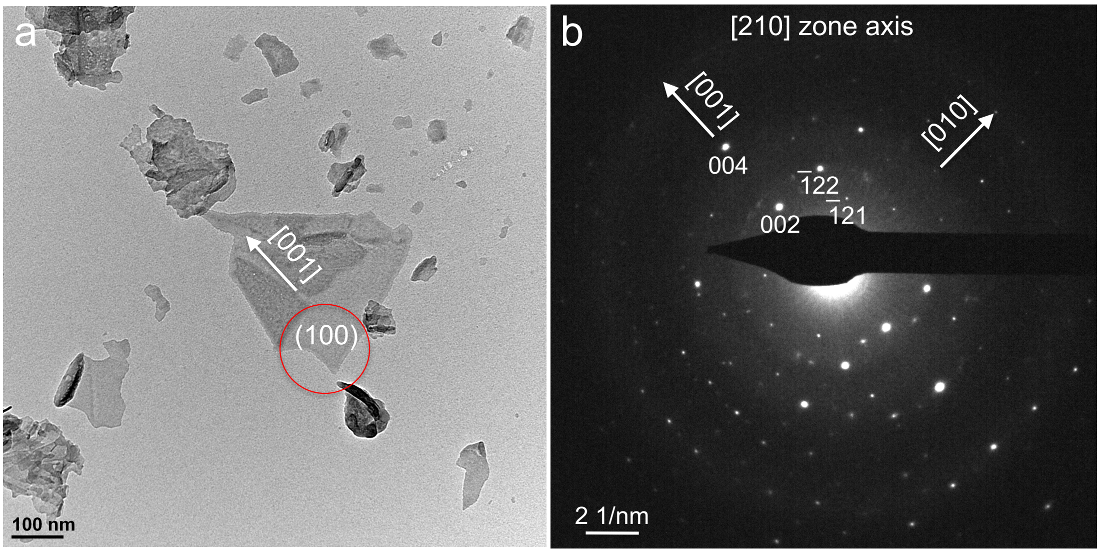


**Figure S9.** TEM-SAED analysis of hydroxylapatite platelets. a) Bright field TEM image of hydroxylapatite crystals formed in the absence of citrate (control run). Note that in addition to platelets, there are a few fiber-like crystals showing a high contrast. They correspond to edge-on platelets. The (*hkl*) indexing on the platelet located in the center corresponds to that determined by SAED. Note that the darker contrast areas in the central platelet are due to either folding-in of the particle edges or piling up of platelets; b) SAED pattern of the red circled area in (a). Indexing of selected diffraction spot and reciprocal lattice directions are indicated (assuming space group $P6_3/m$). The pattern corresponds to the [210] zone axis, which is normal to the (100) plane of hydroxyapatite. Note that the *d*-spacing of the 1120 reflection is the same of the 110 reflection (0.46 nm), while that of the 1122 reflection is equal to that of 112 (0.27 nm) as they correspond to equivalent planes in the hexagonal unit cell. Extra spots are due to the contribution of the edge-on particle at the bottom of the red circle in (a), which was also irradiated by the e-beam.

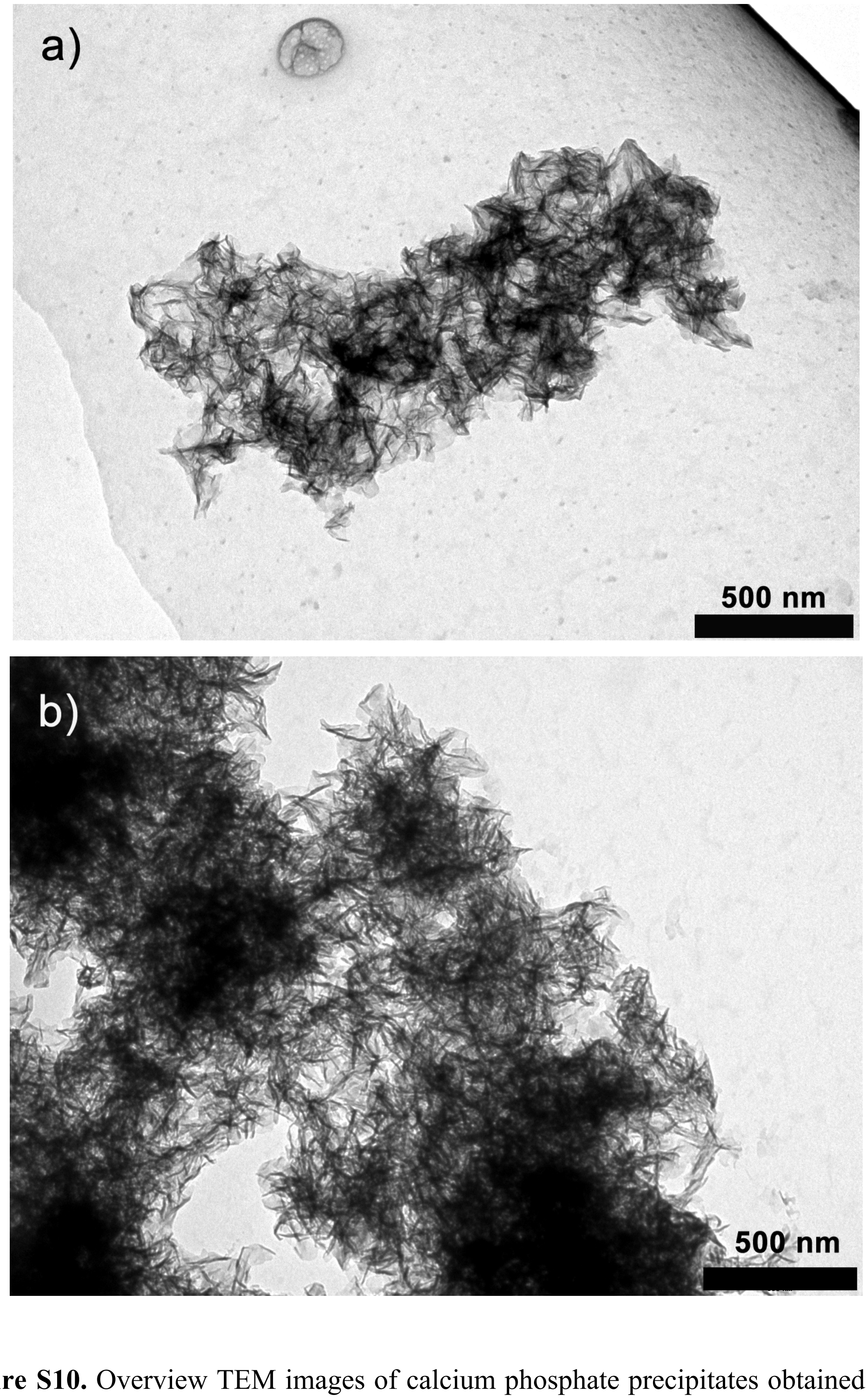


**Figure S10.** Overview TEM images of calcium phosphate precipitates obtained after the titration experiments performed at pH 8: a) 0 (second black arrow in Figure 2) and b) 0.5 mM citrate (second green arrow in Figure 2).

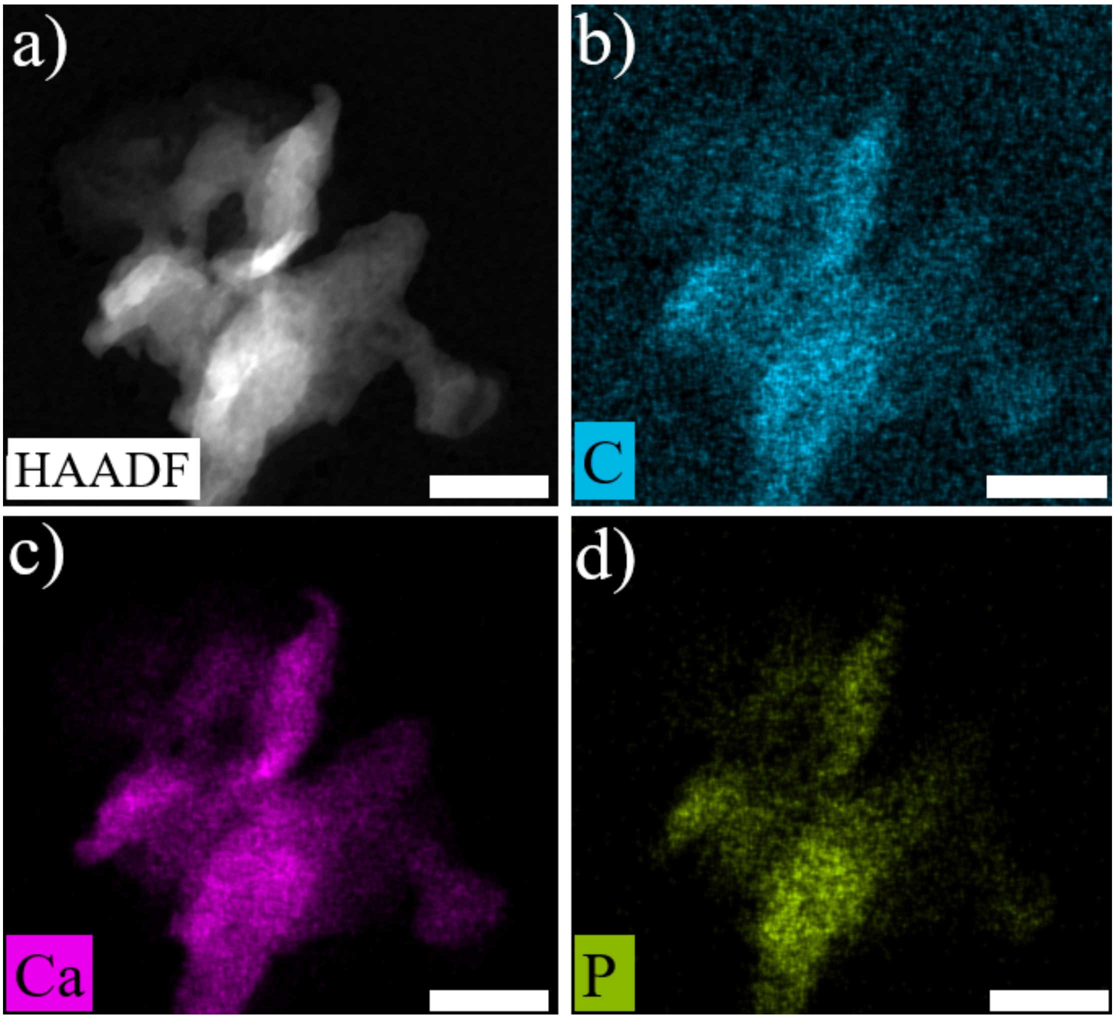


**Figure S11.** a) STEM-HAADF image of HAP precipitates formed in the presence of 0.5 mM citrate at pH 8 (second green arrow in Figure 1b). b) Carbon, c) calcium and d) phosphorus elemental EDS maps of the area in (a). Scale bar 50 nm.

## 2. Application of a multiple binding equilibrium model for estimation of the standard free energy of cluster formation

A simplified multiple binding equilibrium was applied for the linear part of the free-calcium concentration curve in the pre-nucleation regime in order to quantify the standard free energy of cluster formation, $\Delta G_{ion\_pair}$, as in Gebauer et al. (2008)[S1]. The macroscopic equilibrium constant for the formation of calcium/phosphate ion associates, K, can be calculated from:

$$v = \frac{n_{bound}(PO_4^{-3})}{n_{bound}(Ca^{2+})} + \frac{n_{free}(PO_4^{-3})}{n_{bound}(Ca^{2+})} = \frac{1}{x} + \frac{1}{x \cdot K \cdot c_{free}(Ca^{2+})}$$

$c_{free}$ ($Ca^{2+}$), $n_{free}$ ($PO_4^{3-}$), and $n_{bound}$ ($Ca^{2+}$) can be determined from calcium potential measurements. If the macroscopic $\frac{n_{bound}(PO_4^{-3})}{n_{bound}(Ca^{2+})}$ is known, the slope of a plot of v vs. the free calcium concentration, $c_{free}$ ($Ca^{2+}$), gives the reciprocal of the microscopic equilibrium constant, K, for the formation of calcium/oxalate ion pairs and the averaged, dynamic coordination number of a single phosphate, x.

The macroscopic $PO_4^{3-}$ : $Ca^{2+}$ bound ratio can be determined by comparing the concentration of CaP complexes in solution calculated independently from ion-selective electrode (ISE) and conductivity measurements, as in Ruiz-Agudo et al. (2017) [S2]. A theoretical conductivity value can be calculated for the control titration run (i.e., no citrate in the reaction media) from the contributions of all cations and anions in the system, using Kohlrausch's law of independent ion migration for the case of infinite dilution (ideal conditions). This value is higher than the actual measured conductivity, due to the association of ions in solution prior to nucleation. In this work, the best matching between bound phosphate determined from conductivity measurements and the bound calcium (from ISE) was achieved considering a macroscopic $PO_4^{3-}$ : $Ca^{2+}$ ratio in the ion associates, $\frac{n_{bound}(PO_4^{-3})}{n_{bound}(Ca^{2+})}$, of ca. 5:4. This suggests that pre-nucleation associates are negatively charged, as in the case of calcium oxalate, but contrary to other systems such as $CaCO_3$ or $BaSO_4$ in which prenucleation associates are neutral species.

Once the average equilibrium constant for the formation of ion associates in solution (pairs and/or bigger associates) is calculated as indicated above, their standard free energy of formation can be determined as:

$$-RT \cdot lnK = \Delta G_{ion-pair}$$

where R is the gas constant and T the temperature in K. $\Delta G_{ion\_pair}$ relates to the binding strength in clusters. The results of these calculations are shown in Figure S3. From these results, it can be concluded that citrate stabilizes the pre-nucleation

species (i.e., more negative values of $\Delta G_{ion\_pair}$ are found in the presence of citrate). Two additional citrate concentrations (5 and 10 mM) were tested to confirm this trend.

**3. XRD and FTIR identification of precipitates**

Concerning the interpretation of our experimental XRPD diagrams, we adopted the hexagonal symmetry, according to (JCPDS 9-432), for the sake of practice and simplicity. Octacalcium phosphate (OCP, Ca8(HPO4)2(PO4)4·5H2O) and poorly crystalline HAp show similar XRD experimental patterns, due to the structural resemblances among these phases. The presence of the 100 peak of OCP at 2θ= 4.7° ($d_{100}$= 1.878 nm) can be used for distinguishing them based on XRD analyses. In all our experiments, both in the absence and in the presence of citrate, this peak is absent, thereby confirming that the final precipitate is indeed HAP. Moreover, this agrees with the IR spectra of the final precipitates.

The two distinct bands at ca. 1195 and 916 $cm^{-1}$, assignable to $HPO_4^{2-}$ ions in the OCP lattice configuration and thus observed for OCP but not for apatite, are absent in our samples. The 962 ± 2 $cm^{-1}$ band corresponds to $\upsilon_1$-$PO_4$ and is detectable both in apatite and OCP, but not for other calcium phosphate phases. For HAP, there are two vibration bands located at 632 and 3572 $cm^{-1}$ that correspond to $OH^-$ ions in apatitic environments. Nanocrystalline apatites (such as that precipitated in our experiments) show changes in the IR spectra compared to that of stoichiometric, well-crystallized HAP. First, the low degree of crystallinity tends to enlarge vibrational bands, masking weak OH signals. Also, such precipitated apatites are generally associated with water molecules, leading to strong absorption in the 3000–3700 $cm^{-1}$ region. Finally, these precipitated apatites are commonly nonstoichiometric, presenting numerous calcium and hydroxide vacancies; the limited amount of $OH^-$ ions hinders the observation of OH vibration bands by IR spectroscopy. Therefore the 3572 $cm^{-1}$ band is not observed in our samples (at least as a narrow peak). Similarly, in the case of bone apatite, the lack of a sharp band at 3572 $cm^{-1}$ has been interpreted as an indication that bone apatite might not be hydroxylapatite *s.s.*[S3] However, NMR studies show that $OH^-$ are present in small quantities in bone apatite.[S4] Other changes related to the presence of $HPO_4^{2-}$ ions include a broad shoulder typically expanding from 500 to 550 $cm^{-1}$ that is the combination of two components corresponding to non-apatitic and apatitic $HPO_4^{2-}$ and a shoulder around 1145 $cm^{-1}$ (marked by an asterisk in Fig. S5) Finally, a

thin band at 875cm$^{-1}$ is also detected for $HPO_4$-bearing apatites due to the P-OH stretching[S5]. Citrate adsorption and/or incorporation in the precipitates is consistent with the presence of symmetric and asymmetric -$COO^-$ stretching modes at 1417 and 1570 cm$^{-1}$, respectively.

## References


S1 D. Gebauer, A. Völkel, H. Cölfen, *Science* **2008**, *322*, 1819–1822.

S2 E. Ruiz-Agudo, A. Burgos-Cara, C. Ruiz-Agudo, A. Ibañez-Velasco, H. Cölfen, C. Rodriguez-Navarro, *Nat. Commun.* **2017**, *8*, 768.

S3 B. Wopenka, J. D. Pasteris, *Materials Science and Engineering: C* **2005**, *25*, 131.

S4 G. Cho, Y. Wu, J. L. Ackerman, *Science* **2003**, *300*, 1123.

S5 C. Drouet, S. J. Stachelek, *BioMed Research International* **2013**, *2013*, 490946.
